# Relaying Aversive Ultrasonic Alarm Calls Depends on Previous Experience. Empathy, Social Buffering, or Panic?

**DOI:** 10.3390/brainsci11060759

**Published:** 2021-06-08

**Authors:** Wiktoria Karwicka, Marta Wiatrowska, Kacper Kondrakiewicz, Ewelina Knapska, Miron Bartosz Kursa, Adam Hamed

**Affiliations:** 1 Laboratory of Spatial Memory, Nencki Institute of Experimental Biology, Polish Academy of Sciences, 3 Pasteur Street, 02-093 Warsaw, Poland; w.karwicka@nencki.edu.pl; 2 Laboratory of Emotions Neurobiology, BRAINCITY—Centre of Excellence for Neural Plasticity and Brain Disorders, Nencki Institute of Experimental Biology, Polish Academy of Sciences, 3 Pasteur Street, 02-093 Warsaw, Poland; m.wiatrowska@nencki.edu.pl (M.W.); k.kondrakiewicz@nencki.edu.pl (K.K.); e.knapska@nencki.edu.pl (E.K.); 3 Interdisciplinary Centre for Mathematical and Computational Modelling, University of Warsaw, Pawinskiego 5A, 02-106 Warsaw, Poland; m.kursa@icm.edu.pl

**Keywords:** ultrasonic vocalization, social buffering, 50 kHz calls, 22 kHz calls, distress, emotional contagion, fear contagion, aversive state, communication

## Abstract

Ultrasonic vocalizations are among the oldest evolutionarily forms of animal communication. In order to study the communication patterns in an aversive social situation, we used a behavioral model in which one animal, the observer, is witnessing as his cagemate, the demonstrator, is experiencing a series of mild electrical foot shocks. We studied the effect of the foot shock experience on the observer and the influence of a warning sound (emitted shortly before the shock) on USV communication. These experiments revealed that such a warning seems to increase the arousal level, which differentiates the responses depending on previous experience. This can be identified by the emission of characteristic, short 22 kHz calls of a duration below 100 ms. Two rats emitted calls that overlapped in time. Analysis of these overlaps revealed that in ‘warned’ pairs with a naive observer, 22 kHz calls were mixed with 50 kHz calls. This fact, combined with a high fraction of very high-pitched 50 kHz calls (over 75 kHz), suggests the presence of the phenomenon of social buffering. Pure 22 kHz overlaps were mostly found in ‘warned’ pairs with an experienced observer, suggesting a possible fear contagion with distress sharing. The results show the importance of dividing 22 kHz calls into long and short categories.

## 1. Introduction

The ultrasonic vocalizations (USVs) of animals are among the oldest evolutionarily forms of communication [1]. Rats emit sounds varying in frequency that are inaudible to humans and in a band above 20 kHz, reaching a frequency of up to 125 kHz. Most rodent vocal communication research focuses on appetitive sounds—in the so-called “50 kHz” class—that derive from a positive emotional state [2,3]. Ultrasonic vocalizations ranging from 30 to 125 kHz can be induced by addictive substances [4,5,6,7,8], positive social interaction [9,10,11,12,13,14,15], the anticipation of reward [16,17], and in response to a context associated with appetitive conditioning [4,17,18,19,20].

However, rodents, like humans, not only experience and show their positive states but also express negative emotional states [21,22,23]. Jaak Panksepp, an author of the concept of affective neuroscience, classified seven basic emotions as a result of artificial stimulation of the mammalian brain: seeking, rage, fear, lust, care, panic/grief, and play [24].

22 kHz USV calls, with a frequency bandwidth of 18 to 28 kHz, serve as indicators of rats’ negative emotional states, such as distress, discomfort, or anxiety [25,26,27,28]. These aversive types of emissions are usually observed in stressful or endangering situations, such as the presence of a predator [29,30,31,32], the aggressive behavior of other conspecific(s) [26,33,34,35], or when exposed to negative stimuli [36,37,38]. Experiments using playback techniques have shown that exposure to 22 kHz calls elicits freezing and avoidance responses [39,40,41]. Several reports [21,22,35,42] state that 22 kHz calls have a communicative function in a group of rats and are not merely an expression of negative emotions. Litvin et al. (2007) proposed a subdivision of calls based on the situation and purpose of their emission: ultrasonic “warning calls” that are meant to warn conspecifics about danger [39,43] and sonic “alarm calls” that are supposed to discourage predators [29,31,32,43]. In both cases, the calls are risk assessment dependent and appear only if potential benefits outweigh the costs [43]. Interestingly, the documented functions of ultrasound emissions in the 22 kHz class include producing alarm signals to protect the social group [44,45]. Thanks to the modern technology of recording and analyzing USVs, we can determine the animal’s emotional state with increasing precision. Moreover, we can register sounds lasting even a few milliseconds with greater accuracy and resolution. With such tools, we can determine the course of emotional states during behaviorally modulated social interaction.

The neurobiological foundations of ultrasonic vocalizations and their association with the emotional states of rodents are still under investigation. It has been demonstrated that stimulation of cholinergic neurons in the laterodorsal tegmental nucleus triggers 22 kHz calls [46]. Moreover, previous research has shown that the mPFC (medial prefrontal cortex) plays an important role in modulation of 22 kHz calls and that lesion of this structure considerably or completely reduces the number of this type of call [12,47,48]. Dupin et al. (2019) investigated the relationship between electrophysiological data, respiration, and the emission of USVs, suggesting that sequences of USVs could result in a differential gating of information within the network of structures sustaining fear or anxiety behavior. The emission of 22 kHz ultrasonic vocalization calls converges with decreased theta power and increased delta and gamma power in the BLA (basolateral amygdala), the mPFC, and the olfactory piriform cortex (PIR) [49].

In our previous research, we noted occasional 22 kHz signal amplification in pairs of rats. Based on that observation, we aimed to verify the hypothesis that the experience of an electric shock in the past could change the organization of communication between two familiar rats. An in-depth examination of the communication patterns during stressful situations would explain why an observer might behave differently, depending on previous experience.

To study the communicative function of 22 kHz USVs, we used a behavioral model in which one animal, the observer, is witnessing as his cagemate, the demonstrator, is experiencing a series of mild electrical foot shocks [50]. Aversive stimuli in this model elicited 22 kHz vocalizations [50,51]. Previous works have shown that the experience of aversive stimuli may modulate the future empathetic responses of animals [52,53,54]. For instance, previous studies that used the same behavioral model showed that prior experience with foot shocks increases the freezing of the observers [55]. Thus, we compared the vocalizations of the pairs of rats with experienced and inexperienced observers. In addition, in half of the groups, we added the protocol of a 19 s, 1.75 kHz audio signal, which was the foot shock introduction (warning signal). This signal was intended to associate the external stimulus with the demonstrator’s foot shock. Simultaneously, this allowed us to investigate how the prediction of the stimulus affects the behavior of the demonstrator and of the observer.

An essential part of the study design was to exclude the animals’ aversive conditioning response. The electric shocks, intended to familiarize experienced observers with aversive stimuli, were administered in a different spatial context. The training cage differed in shape, lighting, smell, and sound from the one in which we performed the test.

In our study, we aimed to (1) compare USV emissions in pairs of rats between foot-shock-experienced and -inexperienced (naive) observers, (2) examine whether experienced observers amplify the 22 kHz aversive signal emitted by demonstrators, and (3) test whether the warning sound signaling foot shocks changes communication between rats.

## 2. Materials and Methods

### 2.1. Animals

Forty experimentally naive male Wistar rats (250–300 g at the beginning of the experiment) were used in the experiment. The animals were supplied by the Center of Experimental Medicine in Bialystok, Poland. Subjects were randomly paired and housed together in standard home cages (43.0 × 25.0 × 18.5 cm). They were kept in standard laboratory conditions under a 12/12 light–dark cycle and were provided with free access to food and water. All experiments were carried out in accordance with the Polish Act on Animal Welfare, after obtaining permission (126/2016) from the First Warsaw Ethical Committee on Animal Research. 

### 2.2. Experimental Procedure

#### 2.2.1. Habituation

**Figure 1:**
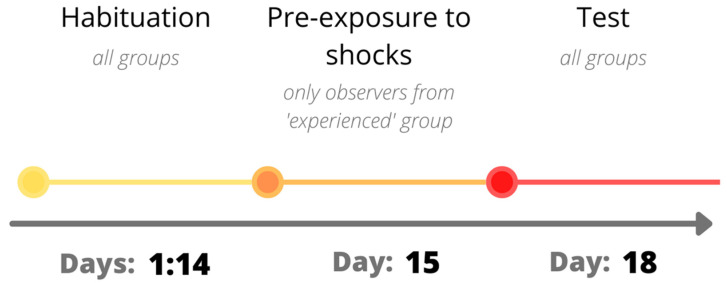
A graphic representation of the experimental design.

After initial acclimatization to a home cage (4 days), the rats were habituated for 10 days to the experimenter’s hand (4–5 min/pair/day) and to the experimental room (20 min for 3 consecutive days in dimmed light), and they were transported between rooms (Figure 1). Pairs of rats were randomly assigned to three experimental groups: control, naive, and experienced. Within each pair, rats were additionally marked either as a demonstrator or as an observer; this division was not necessary for pairs in the control group, as both of the rats underwent the same procedure. The experiment was carried out in two groups—‘warned’ and ‘unwarned,’ with the 3 subgroups that underwent each procedure consisting of the following number of animals:

1. Naive—4 demonstrators and 4 observers;
2. Experienced—4 demonstrators and 4 observers;
3. Control—4 animals, undivided into demonstrators and observers.

#### 2.2.2. Pre-Exposure to Shocks

Three days prior to the experiment, observers from the experienced subgroup were placed into the aversive conditioning cage (Panlab). After 1 min habituation, the rats received 3 electric shocks (with an intensity of 0.7 mA and a duration of 1 s), with a 1 min time interval between each shock. To avoid aversive conditioning to the specific context, the following measures were taken:

- The exposure was performed in a different behavioral room than the following test;
- The cage was illuminated with a bright, white light;
- The interior was sprayed with 1% acetic acid which left a strong smell;
- A plastic rooftop was installed to obtain a triangular shape for different spatial cues.

**Figure 2:**
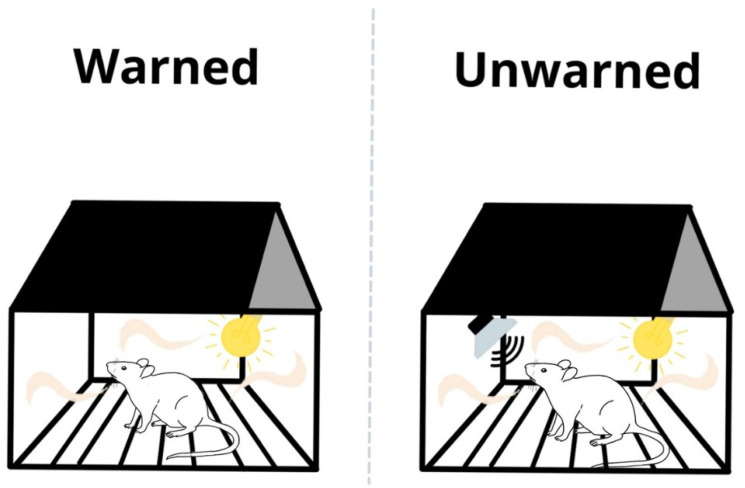
Illustration of the training: pre-exposure to the electric foot shocks. To exclude the rats’ aversive conditioning response, the training cage differed from the test cage in terms of smell, light intensity, and shape. Depending on the experimental group, ‘unwarned’ or ‘warned,’ (the group names are according to the test day procedure), a 1.75 kHz sound signaling a foot shock was emitted or not emitted, respectively.

During conditioning of the unwarned group, a 1.75 kHz sound (19 s duration) was also emitted prior to each shock (Figure 1 and Figure 2).

#### 2.2.3. Test Day

**Figure 3:**
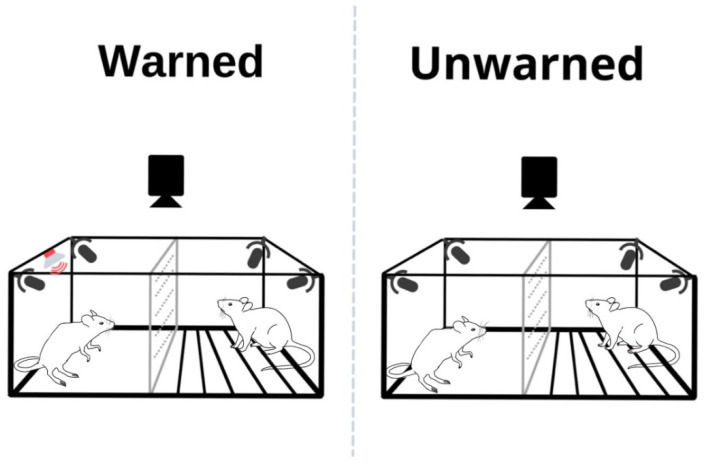
Illustration of the test session. To exclude the rats’ aversive conditioning response, the test cage differed from the training cage in terms of smell, light intensity, and shape. Depending on the experimental group, ‘warned’ or ‘unwarned,’ a 1.75 kHz sound signaling a foot shock was delivered or not delivered, respectively.

On the 18th day of the experiment, the test was carried out in a specially constructed cage (62 × 48 × 25 cm) made of transparent plastic (details in [50]). A perforated, transparent plexiglass divided the interior into two halves—one intended for the observer and the other for the demonstrator. Metal rods installed in the demonstrators’ section were connected to the current generator (MedAssociates), which allowed for the administration of mild electrical shocks. The rats could see, hear, and smell each other throughout the test. During the test, the animals were recorded with a digital camera and with 4 microphones (UltraSoundGate, Avisoft). The test session and the pre-exposure to shocks were conducted in separate rooms. To provide a different context, the lights were dimmed, and the cage was cleaned with a 1% acetone solution after testing each pair. Two minutes after inserting the rats into the appropriate chambers, the demonstrator was given a series of 10 foot shocks (1 s, 1.0 mA). In the ‘warned’ group, each electric shock was signaled by a 19 s, 1.75 kHz sound, equaling a total of 80 s between 2 shocks, whereas in the ‘unwarned’ group, there was no sound indication and the shocks were administered in a 1 min interval (Figure 1 and Figure 3).

### 2.3. Apparatus and USV Recordings

USVs were recorded using an UltraSoundGate Condenser Microphone CM16 (Avisoft Bioacoustics, Berlin, Germany) that was positioned 25–30 cm above the floor of the cage. The microphone was sensitive to frequencies of 15–180 kHz with a flat frequency response (±6 dB) between 25 and 140 kHz. The microphone was connected to an amplifier (custom-made, Warsaw) that had the following parameters: a voltage gain of 16 *v*/*v* (12 dB), a frequency response of ±0.1 dB, a range of 30 Hz to 120 kHz, and an input impedance of 600 Ω. The signal was then transferred through a 120 kHz anti-aliasing filter (custom-made, Warsaw). The filtered sounds were sent to a PCI-703-16A data acquisition board (Eagle Technology, Chicago, IL, USA). This board was a 14 bit, 400 kHz analogue input and output board for PCI-based systems. The recorded data were processed using the RAT-REC PRO 7.3 software (custom-made, Warsaw, Poland). The signals were processed through a fast Fourier transformation (1024, Hamming or Hann window) and displayed as color spectrograms. Each signal was manually marked with the section label included in the automated parameter measurement.

Two rats from each pair were recorded simultaneously. All vocalizations from calls that overlapped in time (occurrence of the two vocalizations at the same time) were marked as separate episodes and analyzed individually. All harmonic sounds were excluded; only fundamental frequencies, which indicate at most two actual calls in every case, were analyzed. Various parameters were determined automatically, including the number of USV calls, the total calling time (s), the mean call duration (s), the frequency bandwidth (kHz), the number of gaps, the mean gap duration (s), and the mean peak frequency (kHz). The signal from the microphone was sent to another room where the computer and research observer were situated (more in [4,10,11]). 

### 2.4. Statistics

To assess the relationship between continuous and categorical variables, we used the Kruskal–Wallis test, followed by the Conover–Iman post-hoc test for the identification of precise pairwise differences, but only when the result of the Kruskal–Wallis test was significant. A significance level of 0.05 and two-sided testing were employed. All statistical analyses were performed in R, version 4.0.5, using the conover.test package version 1.1.5.

## 3. Results

### 3.1. The Effects of the Warning Signal. Fractions of the Ultrasonic Vocalizations during Social Communication in the Social Transfer of Fear Paradigm

**Figure 4:**
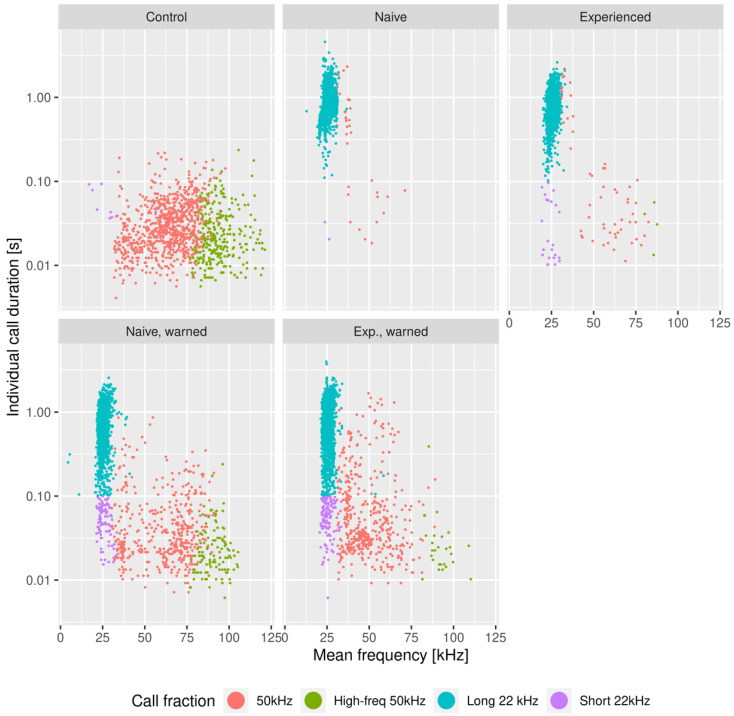
Scatterplot of the basic properties of the recorded USV calls, illustrating their mean frequency and duration. Each panel collects all vocalizations recorded for pairs of a certain class. Each of the presented groups were represented by four pairs of rats (eight animals per group). Color denotes the call fraction. Note that the duration is shown on a logarithmic scale.

Figure 4 presents the joint distribution of the duration and frequency of every call considered in the paper, split into experimental groups. The various fractions of calls are clearly visible, so there are quantitative and qualitative differences between them across the groups. The 22 kHz fraction is strongly present in all groups, except in the control group. In ‘unwarned’ groups, however, its duration span is substantially limited, which corresponds to a lack of episodes, which we later refer to as “short 22 kHz” calls.

**Figure 5:**
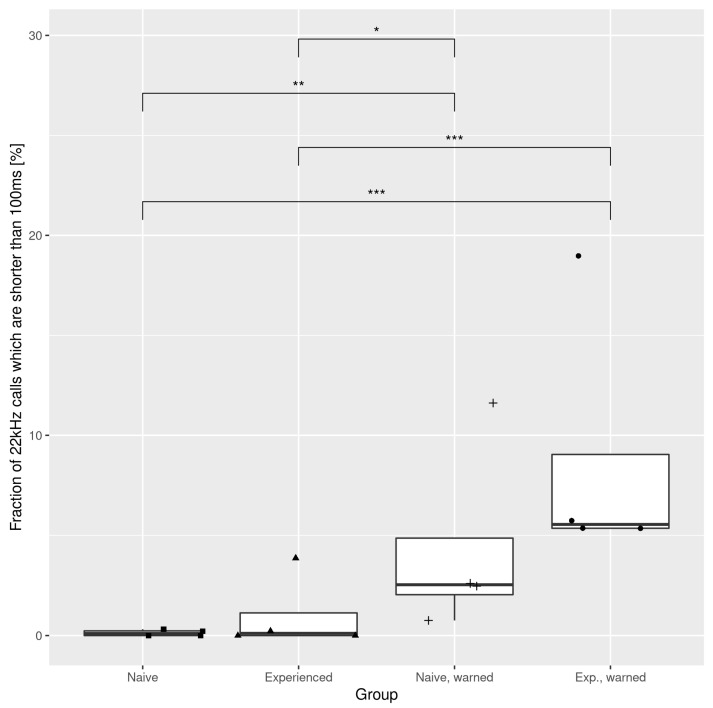
Fraction of short (<100 ms) 22 kHz calls in the different experimental groups. Brackets mark significant differences identified by the Conover–Iman test. * *p* < 0.05; ** *p* < 0.01; *** *p* < 0.001.

Figure 5 shows the quantitative differences between short 22 kHz calls in the different groups together with the results of the Conover–Iman test. One can see that short 22 kHz calls are especially abundant in the ‘experienced, warned’ class, and significantly more numerous than in either of the ‘unwarned’ groups; similarly, the ‘naive, warned’ group emitted a significantly increased number of short 22 kHz calls than the aforementioned ‘unwarned’ groups. 

**Figure 6:**
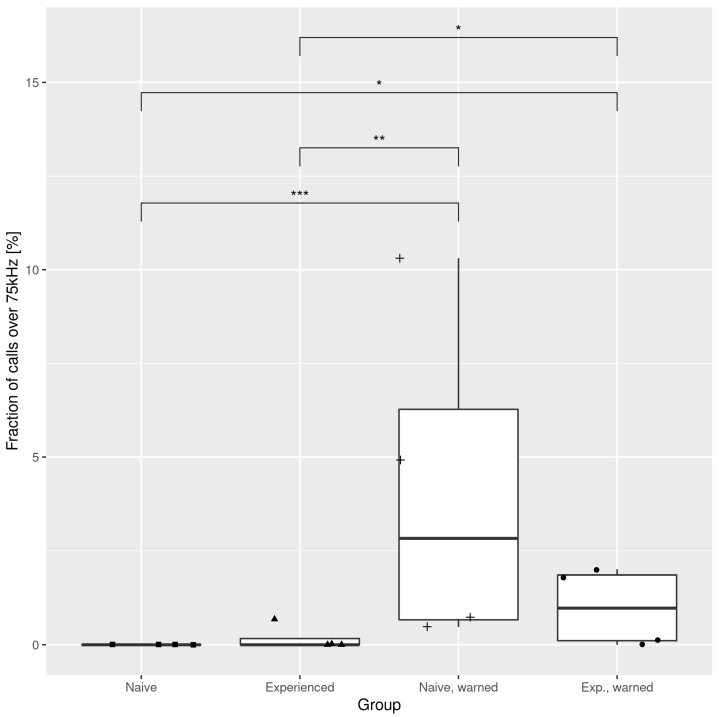
Fraction of 50 kHz calls with a frequency above 75 kHz in different experimental groups. Brackets mark significant differences identified by the Conover–Iman test. * *p* < 0.05; ** *p* < 0.01; *** *p* < 0.001.

Returning to Figure 4, one can note that the 50 kHz calls build a sparse cluster with a wide span in both parameters. Again, it is less evident in the ‘unwarned’ groups. The center of the 50 kHz cluster also varies between groups, which is reflected in the average frequency, as well as in the abundance of 50 kHz calls above the 75 kHz boundary, which we later refer to as “high-frequency 50 kHz” calls (Figure 6).

**Figure 7:**
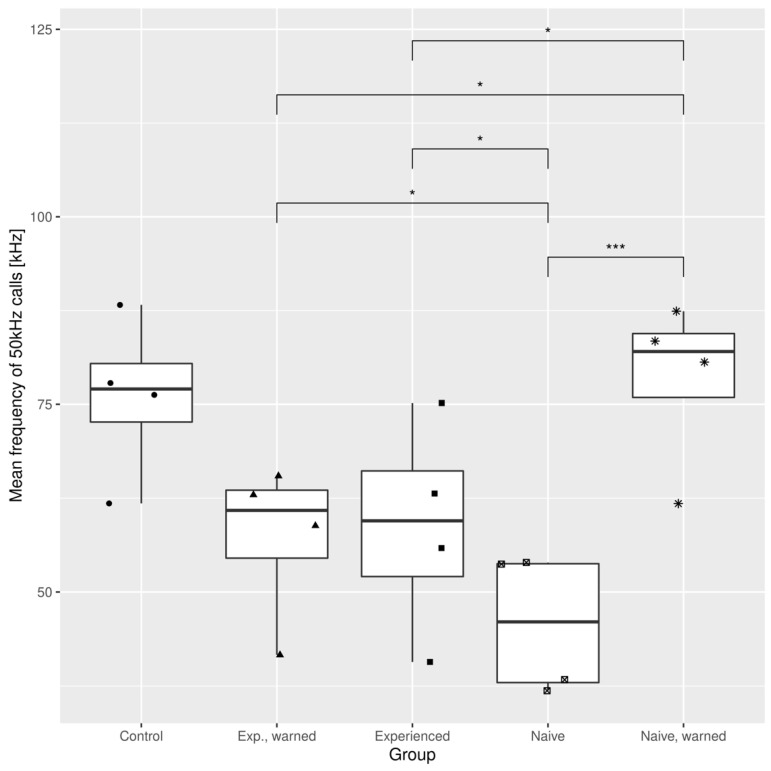
Mean frequency of 50 kHz calls in different experimental groups. Brackets mark significant differences identified by the Conover–Iman test. * *p* < 0.05; *** *p* < 0.001.

Figure 6 shows the comparison of high-frequency 50 kHz calls between groups. As with the fraction of short 22 kHz calls, each of the ‘warned’ groups exhibited a significantly higher abundance of these 50 kHz calls than either of the ‘unwarned’ groups. The highest fraction of high-frequency calls was found in the ‘naive, warned’ group, which formed over 10% of all calls. The same conclusions can be drawn from Figure 7, which presents the mean frequencies of 50 kHz calls in each pair. Here, the ‘naive, warned’ group generated the highest pitched calls of all the experimental groups, with a median over 75 kHz. This result was similar to that achieved by the control pairs. Beyond those findings, we can also see that among ‘unwarned’ groups, ‘experienced’ rats produced higher USVs than ‘naive’ ones.

### 3.2. The Effects of the Warning Signal. Overlapping of the Ultrasonic Vocalizations during Social Communication in the Social Transfer of the Fear Paradigm

**Figure 8:**
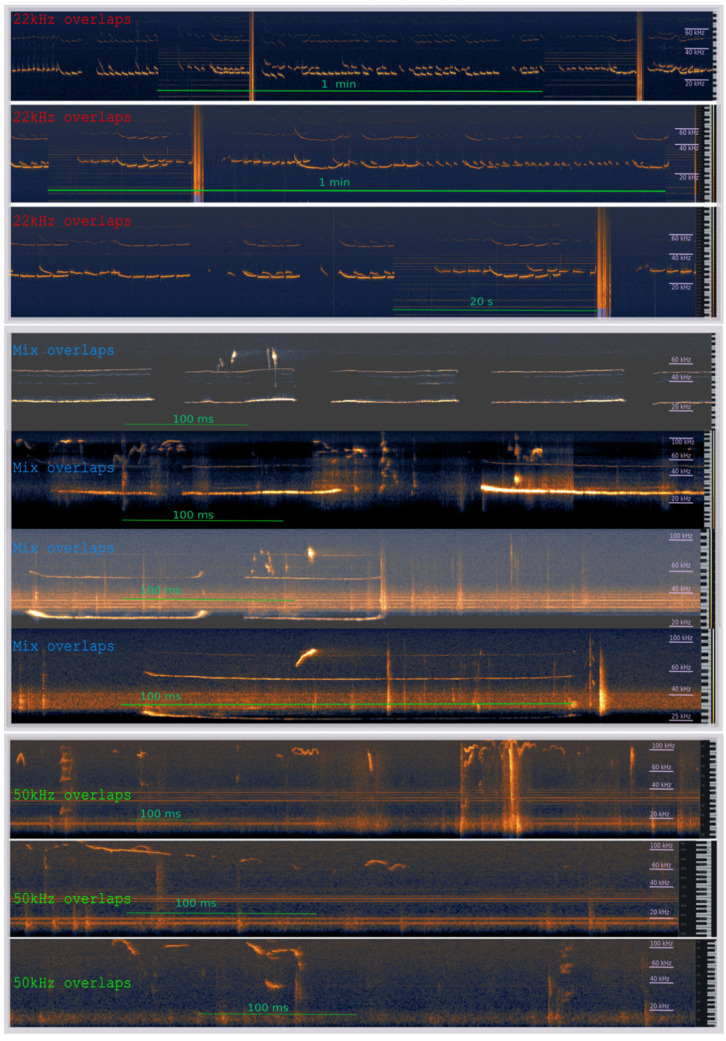
An example of overlapping ultrasonic vocalization presented on a spectrogram.

**Figure 9:**
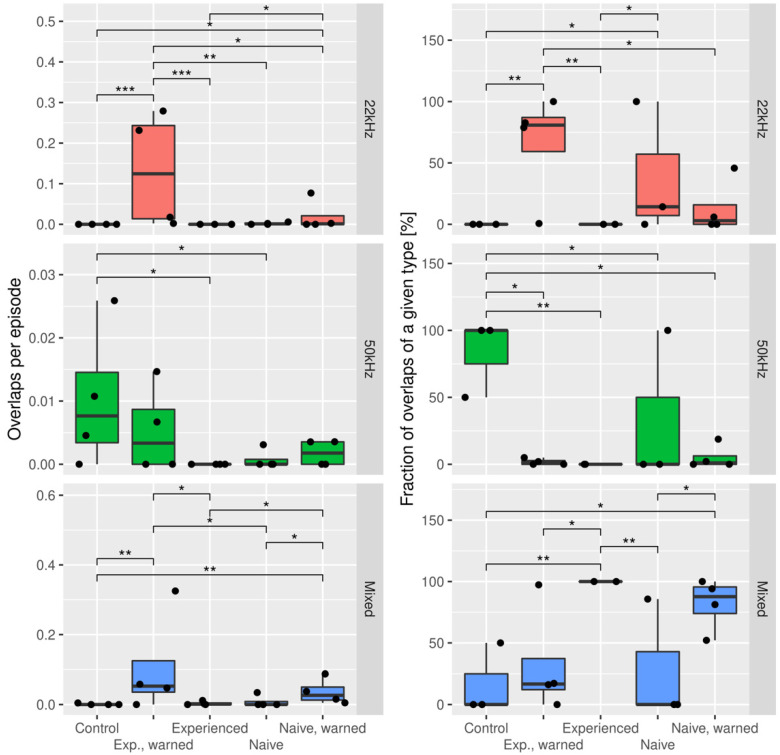
Fractions of temporal USV overlaps which belong to a certain class: 22 kHz—two 22 kHz calls overlapping, 50 kHz—two 50 kHz calls overlapping, mixed—22 and 50 kHz calls overlapping in different experimental groups. The left panels show the count normalized by the episode count, while the right panels show the count normalized by the total count of temporally overlapping USVs. Brackets mark significant differences identified by the Conover–Iman test. * *p* < 0.05; ** *p* < 0.01; *** *p* < 0.001.

Some of the reported USV calls were temporally overlapping (these overlapping calls originated from the vocalizations of two rats at the same time); that is, one coherent signal could be seen as superimposed on another, as can be seen in Figure 8. We extracted all such events and denoted the frequencies of each of the two involved vocalizations. Using this key, overlaps can be divided into three types: a pair of 22 kHz calls, a pair of two 50 kHz calls, and an overlap of 22 and 50 kHz calls, which we later refer to as a mixed overlap. Figure 9 reports the distribution of said overlap types, both in relation to all recorded calls and to the overall overlapping USVs.

One can see that ‘experienced, warned’ rats had a substantial number of pure 22 kHz overlaps, up to almost 3 per 10 episodes in the case of 1 pair. This is significantly higher than in any other experimental group.

In the ‘naive, warned’ rats, the overlap rate was smaller than in the ‘experienced, warned’ rats, but larger than in either of the ‘unwarned’ experimental groups. In relative terms, the mixed overlaps constituted from approximately 50% to 100% of all overlaps identified in this group.

Finally, in the control group, the most substantial type of overlaps were pure 50 kHz calls.

## 4. Discussion

In this study, we conducted a detailed analysis of the ultrasonic vocalizations emitted by pairs of rats during two slightly different aversive situations. In one of the groups, the electrical shocks were signaled by the emission of a 19-s audible (warning) sound (‘warned’ group), while the other experimental group did not receive any external acoustic signals before the electrical stimulus (‘unwarned’ group). The tested animals either had previous experience with foot shocks (‘experienced’ group) or did not have such experience (‘naive’ group). We were interested in whether there was a difference in communication patterns between ‘warned’ and ‘unwarned’ animals and whether the experience of the electrical stimulation would change the animals’ behavior in an aversive situation. The warning sound emitted before the electrical stimulus seemed to increase arousal level, which differentiated the responses, depending on previous experience. 

In the animals from the ‘warned’ group, we identified a fraction of short calls in the “22 kHz” class, which was practically absent in the ‘unwarned’ animals. It has been previously suggested [26,56] that such calls are a sign of distress (internal negative emotional state) rather than a response to external, aversive stimuli triggering the common, longer 22 kHz calls. Our observations provide additional, substantial evidence in favor of this hypothesis. This could mean that the anticipation of electric shock, induced by the warning signal, leads to a higher stress level in contrast to unpredictable shocks, which is in line with the results of fear conditioning studies [57].

Many studies have shown that USV playback or synthetic sound presentation in the 22 kHz class activates the perirhinal cortex, periaqueductal grey matter, the amygdala, or the hypothalamus—the structures involved in defensive behavior [41,58,59]. The ultrasonic vocalizations produced by rats in a stressful or threatening situation may be crucial for their conspecifics’ survival or wellbeing [43]. Both 22 kHz calls [56] and freezing behavior [60] can be interpreted as a warning and used by the observers to learn about danger. Importantly, the reception of the 22 kHz calls changes the function of the brain fear circuit, which probably helps the recipients to quickly adapt to the threatening situation. For example, Dupin et al. (2019), who investigated the relationship between the emission of USVs, respiration, and the electrophysiological activity of brain structures, showed that 22 kHz calls result in a decrease in theta power and an increase in delta and gamma power in the BLA, the mPFC, and the piriform olfactory cortex (PIR)—the structures involved in fear responses [49]. Intense fear, observed as a panic attack, is associated with hyperventilation and breathlessness [61], which can be reduced by paroxetine, a selective serotonin reuptake inhibitor [62]. Interestingly, Willadsen et al. (2020) documented that animals lacking the serotonin transporter emitted a lower number of 22 kHz USVs [63]. These results, together with the correlational studies demonstrated by Dupin et al. (2019), indicate that the animals warned by the audible sound in our research (the ones emitting short 22 kHz signals) were in intense distress. This indicates the crucial role of 22 kHz USVs in emotional contagion within a social group.

We detected a difference between the ‘experienced’ and ‘naive’ groups in the distribution of the frequency of the emitted ultrasonic vocalizations. 

The simplest form of empathy, which can be observed in animals, is defined as the ability to understand or to share another individual’s emotional state [64]. It plays an essential role in regulating social behavior and can be modulated by prior experience [65]. An interesting phenomenon observed in our study is that short 22 kHz episodes that reflect distress are emitted both by the demonstrator and by the observer, as reflected in overlapping 22 kHz calls. Additional intensification of the ultrasound signal in the 22 kHz class appears shortly after the initial short 22 kHz calls, thus indicating emotional contagion; the observer is being “infected” with the demonstrator’s emotional state [51]. Presence of the short 22 kHz USVs indicates accelerated breathing (tachypnea) that is typically related to hyperventilation, which is characteristic of panic states. Henceforth, the occurrence of the same 22 kHz calls in the demonstrator and in the observer indicates that distress may be shared by the pair of rats.

On the other hand, we noted an interesting fraction of very high-frequency 50 kHz calls (over 75 kHz) that were present almost exclusively in the ‘naive, warned’ experimental group. We believe this indicates that naive rats are more inclined to emit soothing calls to reduce the distress of their partners, as explained by the social buffering phenomenon [64,66]. It is known that parallel to fear transfer from the demonstrators to the observers, the observers may provide social support and a moderate stress response to demonstrators, a phenomenon known as social buffering [64]. It has been shown that social buffering is more effective among familiar animals [67]. Furthermore, naive animals are more effective in social buffering than animals subjected to fear conditioning [66,67].

While we were not able to unambiguously attribute each call to the individual rat, we relied on a phenomenon of temporally overlapping calls, which we assumed came from either rat at a particular time. This allowed us to elaborate on their interactions. In particular, we observed all of the possible overlap classes: two 22 kHz calls, two 50 kHz calls, and a mixture of both. 

## 5. Conclusions

Analysis of USV communication patterns reveals that in the presence of an experienced observer, most of the overlapping ultrasonic vocalizations covered a 22 kHz class (Figure 7). In the case of the ‘naive, warned’ observer, we noticed that USV overlaps are mixed signals, including 22 and 50 kHz calls (Figure 7). Moreover, the ultrasounds from the 50 kHz class in the ‘naive, warned’ group mostly consist of calls over 75 kHz (Figure 4, Figure 6 and Figure 8).

Detailed analysis of the USV communication patterns has shown that it is critical to divide the 22 kHz calls into long and short classes in order to precisely quantify emotional processing in rats. In addition, for future studies, we suggest extracting the 50 kHz fraction and dividing it into low- and high-frequency 50 kHz using a 75 kHz threshold, or analyzing their dominant frequency distributions. Currently, the typical 50 kHz analysis approach distinguishes a whole range of subtypes based on their spectrographic shape but without simultaneously considering their frequency. We strongly believe that ultrasonic vocalizations have a prosodic character, which carries the most essential information in social communication, and that the division into specific sub-episodes, distinguished by the different shapes reflected in the FFT, should be taken into account as a second factor.

## Data Availability

Data available upon request.
